# Cryopreservation and xenografting of human ovarian fragments: medulla decreases the phosphatidylserine translocation rate

**DOI:** 10.1186/s12958-016-0213-6

**Published:** 2016-11-10

**Authors:** Vladimir Isachenko, Plamen Todorov, Evgenia Isachenko, Gohar Rahimi, Bettina Hanstein, Mahmoud Salama, Peter Mallmann, Andrey Tchorbanov, Paul Hardiman, Natalie Getreu, Markus Merzenich

**Affiliations:** 1 Research Group for Reproductive Medicine and IVF-Laboratory, Department of Obstetrics and Genecology, Cologne University, Kerpener Str. 34, 50931 Cologne, Germany; 2 Institute of Biology and Immunology of Reproduction, Tzarigradsko shosse 73, 1113 Sofia, Bulgaria; 3 Laboratory of Experimental Immunology, Institute of Microbiology, Acad. G. Bonchev Street, Block 26, 1113 Sofia, Bulgaria; 4 Institute of Women’s Health, University College London, London, UK; 5 MedEvent Dr. Merzenich GmbH, Im Zollhafen 12, 50678 Cologne, Germany

**Keywords:** Cancer, Cryopreservation, Human ovarian tissue, Phosphatidylserine translocation, Medulla, Xenotransplantation

## Abstract

**Background:**

Phosphatidylserine is the phospholipid component which plays a key role in cell cycle signaling, specifically in regards to necrosis and apoptosis. When a cell affected by some negative factors, phosphatidylserine is no longer restricted to the intracellular side of membrane and can be translocated to the extracellular surface of the cell. Cryopreservation can induce translocation of phosphatidylserine in response to hypoxia, increasing intracellular Ca^2+^, osmotic disruption of cellular membranes, generation of reactive oxygen species and lipid peroxidation. As such the aim of this study was to test the level of phosphatidylserine translocation in frozen human medulla-contained and medulla-free ovarian tissue fragments.

**Methods:**

Ovarian fragments from twelve patients were divided into small pieces of two types, medulla-free cortex (Group 1, *n* = 42, 1.5–3.0 × 1.5–3.0 × 0.5–0.8 mm) and cortex with medulla (Group 2, *n* = 42, 1.5–3.0 × 1.5–3.0 × 1.5–2.0 mm), pre-cooled after operative removal to 5 °C for 24 h and then conventionally frozen with 6 % dimethyl sulfoxide, 6 % ethylene glycol and 0.15 M sucrose in standard 5-ml cryo-vials. After thawing at +100 °C and step-wise removal of cryoprotectants in 0.5 M sucrose, ovarian pieces were xenografted to SCID mice for 45 days. The efficacy of tissues cryopreservation, taking into account the presence or absence of medulla, was evaluated by the development of follicles (histology with hematoxylin-eosin) and through the intensity of translocation of phosphatidylserine (FACS with FITC-Annexin V and Propidium Iodide).

**Results:**

For Groups 1 and 2, the mean densities of follicles per 1 mm^3^ were 9.8, and 9.0, respectively. In these groups, 90 and 90 % preantral follicles appeared morphologically normal. However, FACS analysis showed a significantly decreased intensity of translocation of phosphatidylserine (FITC-Annexin V positive) after cryopreservation of tissue with medulla (Group 2, 59.6 %), in contrast with tissue frozen without medulla (Group 1, 78.0 %, *P* < 0.05). In Groups 1 and 2 it was detected that 21.6 and 40.0 % cells were viable (FITC-Annexin V negative, Propidium Iodide negative).

**Conclusion:**

The presence of medulla in ovarian pieces is beneficial for post-thaw development of cryopreserved human ovarian tissue.

## Background

One of the major death causes in the world is cancer. Incidences of cancer are increasing worldwide. In the USA alone, 1,685,210 new cancer cases and 595,690 cancer deaths are projected to occur in 2016 [1]. Furthermore the overall incidence rate for cancer in children aged 14 years and younger increased by 0.6 % per year between 1998 and 2007 [2].

A similar trend has been observed in Europe. Every year in Germany, around 800 girls under age 15 are diagnosed with cancer [3]. At the same time, increased survival rates were observed for all categories of childhood cancers studied, with the extent and temporal pace of the increases varying by diagnosis [4].

Fortunately, recent progress in cancer therapies has significantly decreased mortality rates. However, these therapies whilst often curative often cause significant side effects, including reduced fertility and sterility. For young women surviving cancer this is often a major concern [4]. As such, cryopreservation of ovarian tissue before cancer therapy with re-implantation after convalescence could offer a viable and more successful alternative to current fertility preservation option [5, 6].

Since 1998 several cases reporting restoration of ovarian function after re-implantation of ovarian cortex in patients with premature ovarian failure after cryopreservation of ovarian tissue before the cancer treatment have been published. Furthermore, over 60 live births following cryopreservation and retransplantation of frozen ovarian tissue have been reported [7].

The intracellular side of the cell membrane is made up of phosphatidylserine, the phospholipid component that plays a key role in cell cycle signaling, necrosis and apoptosis. When conditions change and hypoxia is induced, phosphatidylserine is can be translocated to the extracellular surface of the cell and is not just restiructed to intracellular membrane. When this occurs it signals the macrophages to engulf the cells [8].

There are at least five factors that are observed during cryopreservation which induce translocation of phosphatidylserine: hypoxia, increasing of intracellular Ca^2+^, osmotic disruption of cellular membranes, generation of reactive oxygen species (ROS) and lipid peroxidation.

As usual research groups remove the medulla from the cortex when preparing the ovarian tissue for cryopreservation as the cortex contains the majority of the follicles. However, there is still a significant number of follicles within the medulla [9] and most importantly, blood vessels. As such we postulate that keeping the medulla may support neovascularisation in re-implanted tissues.

Consequently, the aim of this study was to compare the viability of follicles in cryopreserved medulla-containing and medulla-free human ovarian cortex by evaluating the intensity of the phosphatidylserine translocation after thawing and xenografting.

## Methods

This study was approved by the Ethics Boards of Universitiy Cologne (applications 99184 and 13–147). Manipulations with SCID mice were approved by the Animal Care Commission at the Institute of Microbiology, Sofia, Bulgaria.

Written informed consents were obtained from all participants aged 18 and over involved in our study. On the behalf of the patients under the age of 18 written consents were obtained from the next of kin.

Except where otherwise stated, all chemicals were obtained from Sigma (Sigma Chemical Co., St. Louis, MO, USA).

### Tissue collection, dissection, and distribution into groups

Informed consent was obtained from 12 patients aged between 13 and 36 (25.1 ± 4.0) years. According to our approved protocol 10 % of ovarian tissue collected from patients was used for ‘patient- oriented’ research. This refers to research which is done in order to assess the viability of the tissue for re-transplantation.

The medium used for transport and dissection, the basal medium, was comprised of Leibovitz L-15 with 5 % Dextran Serum Substitute (Irvine Scientific, Santa Ana, CA, USA).

Following collection the fresh ovarian tissue fragments were transported to the laboratory within 20 min of surgery. The temperature of the sample was maintained between 32 and 34 °C. Using tweezers and scalpel No 22, ovarian fragments were dissected into small pieces (Group1, medulla-free fragments: 1.5–3.0 × 1.5–3.0 × 0.5–0.8 mm and Group 2, medulla-contained fragments: 1.5–3.0 × 1.5–3.0 × 1.5–2.0 mm). Then pieces were cooled at 5 °C for 24 h and on the next day frozen as described below.

In total forty-four pieces, corresponding to two pieces per mouse, in each experimental group, were used to determine the quality of follicles and the degree of phosphatidylserine translocation.

### Tissue cryopreservation (freezing and thawing)

This procedure was performed as published previously [10].

Ovarian tissue was frozen in Germany, then transported, thawed and transplanted to SCID mouse in Bulgaria.

On the day of freezing, pieces of ovarian tissue were placed for 30 min at room temperature in 20 ml freezing medium composed of basal medium supplemented with 6 % dimethyl sulfoxide, 6 % ethylene glycol and 0.15 M sucrose. Then pieces were put into a standard 5-ml cryo-vials (Thermo Fisher Scientific, Rochester, NY, USA) previously filled by 4.5 ml freezing medium and frozen in a IceCube 14S freezer (SyLab, Neupurkersdorf, Austria). The slow cooling profile started at -6 °C, the samples were then cooled from −6 to −34 °C at a rate of −0.3 °C/min. At −34 °C cryo-vials were finally plunged into liquid nitrogen and stored until thawing. The freezing protocol for cryopreservation of this ovarian tissue included auto-seeding step at −6 °C.

In order to thaw the samples, cryo-vials were removed from liquid nitrogen and held for 30 s at room temperature, they were then immersed in a 100 °C (boiling) water bath for 60 s. The exposure time in the boiling water was visually controlled by the presence of ice in the medium; as soon as the ice was 2 to 1 mm apex, the cryo-vial was removed from the boiling water, at which point the final temperature of the medium was between 4 and 10 °C. Within 5–10 s after thawing, the pieces from the cryo-vials were transferred to a 10 ml thawing solution (basal medium containing 0.5 M sucrose) in a 100 ml specimen container (Sarstedt, Nuembrecht, Germany). For stepwise dilution of cryoprotectants, the container was placed on a shaker and continuously agitated with 200 osc/min for 15 min at room temperature.

This was performed using the same, previously published [10] ‘dropping’ methodology: slow addition of basal medium to the solution of sucrose with ovarian pieces. For ‘dropping’, we used 50 ml of basal medium in a 50 ml tube (Greiner Bio-One GmbH, Frickenhausen, Germany). The final sucrose concentration was 0.083 M, resulting in almost isotonic conditions [10]. The last step involved three washes in basal medium for 10 min immediately prior to transplantation into SCID-mice.

### Xenografting of ovarian tissue

Twenty-two, female, 7 week SCID (BALB/c) mice were obtained from Harlan Farm (Blackthorn, UK). The animals were kept in specific- pathogen-free (SPF) conditions with equal 12-h light and dark conditions and provided with food and water ad libitum.

**Fig. 1:**
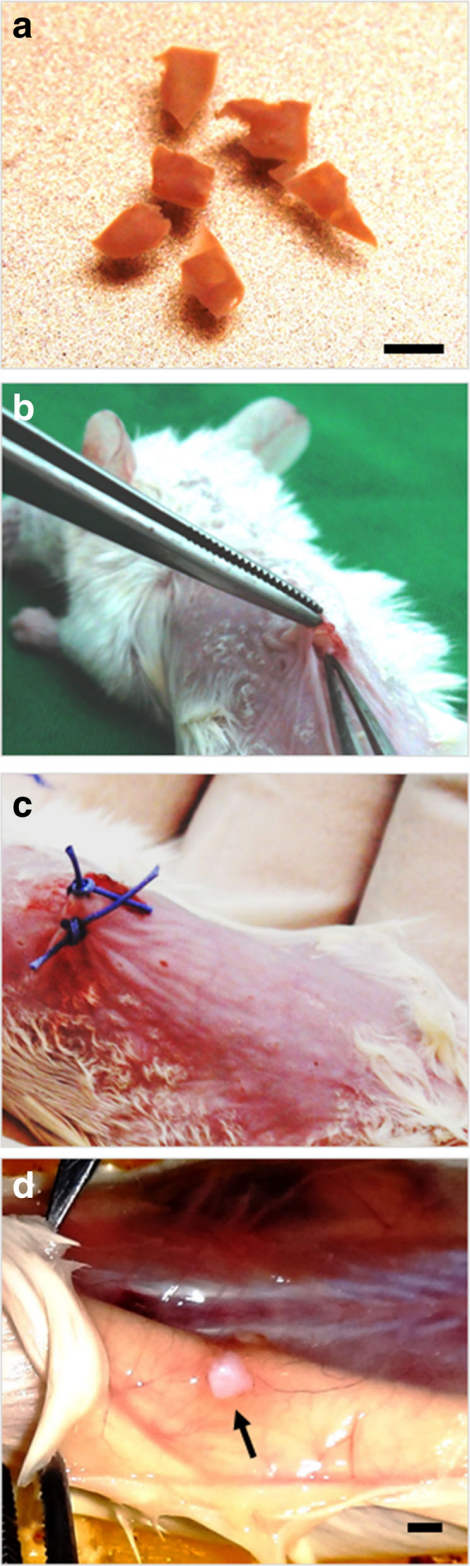
Xenografting of cryopreserved human ovarian tissue. **a** Cryopreserved ovarian pieces just after thawing, **b**, **c** Transplantation of these pieces, **d** Ovarian piece after 45 d in vivo culture. Scale bar: 2 mm

Immediately following thawing, ovarian pieces were transplanted to SCID mice as previously described [11–13] (Fig. 1). During surgery, the mice were kept on a warming plate and the procedure was carried out under sterile conditions. A combination of anesthetic Rompun 2 % (Bayer Vetal Leverkusen, Germany) and Ketanest 50 mg/ml (Parke-Davis Freiburg, Germany) was used for sedation. In each mouse the tissue pieces were grafted dorsolateral on the right and left side of the vertebral column. Tissue from the same patient was used in parallel to the group with medulla and the group without medulla. Every second day the mice were injected with 1.0 IU of recombinant FSH (Gonal-F^®^, Laboratories Serono S.A., Aubonne, Switzerland). The animals were killed by cervical dislocation on the 45th day after transplantation. The recovered grafts were dissected into two parts: the first processed for measurement of phosphatidylserine translocation and the second fixed for histological investigation.

### Phosphatidylserine translocation: flow cytometry

The human tissue was recovered from the mice and replaced to PBS with 10 % fetal calf serum (FCS). Then the tissue was incubated in GM 501 Collagenase (Gynemed GmbH, Lensahn, Germany) for 1 h at 37^0^C while being continually shaking. At the end of the hour the reaction was stopped by addition of ice cold PBS-FCS, followed by mechanical disruption of the tissue through 20G syringe needle and passing through 70 μm filter on ice. The cells were centrifuged at 1000 RPM at 4^0^C for 10 min in PBS-FCS and then suspended in 1 ml PBS-FCS.

The isolated cells were cultured in complete RPMI 1640 (Roswell Park Memorial Institute medium, Gibco, Gaithersburg, MD, USA) containing 10 % FCS, 4 mM L-glutamine, 50 μM 2-mercaptoethanol and antibiotics for 1 h at 37 °C in 5 % CO_2_. Then cells were collected, washed and stained with a FITC-Annexin V apoptosis detection Kit I (BD Biosciences, San Diego, CA, USA), containing Propidium Iodide (PI) as DNA-binding dye in accordance with instruction of manufacturer. The apoptosis of gated cells was analyzed within 15 min. using flow cytometry (BD LSR II flow cytometer). The rate of phosphatidylserine translocation as detector of early apoptosis (FITC-Annexin V positive, PI negative) and late apoptosis and dead cells (FITC-Annexin V positive, PI positive), as well as necrosis (FITC-Annexin V negative, PI positive) was measured.

### Histology of follicles

For histological investigation, the grafted tissue pieces were fixed in Bouin’s solution, embedded in paraffin wax, serially sectioned at 4 μm, stained with hematoxylin/eosin, and analyzed under a microscope (×400, Olympus Co, Tokyo, Japan).

The number of viable and damaged follicles was counted. Before counting of follicles, sections were coded and scored “blind”. To avoid over-counting the same follicles, only sections with a visible oocyte nucleus were analysed. Two types of preantral follicles were evaluated: 1) primordial follicles composed of an oocyte surrounded by a layer of flattened follicular cells and 2) primary and secondary follicles which are similar to primordial follicles, but in which the oocyte is surrounded by one to two layers of spheroid granulosa cells.

Morphology of the follicles was evaluated on the basis of parameters previously described [14]. Here we have slightly modified the mentioned [14] description. The quality of follicles was graded on a scale from one to three. A follicle of grade 1 is spherical in shape and contains a spherical oocyte which is surrounded by an even distribution of granulosa cells and has a homogenous cytoplasm and slightly granulated nucleus, with condensed chromatin in the form of a dense spherical structure detectable in the center of the nucleus. A follicle of grade 2 has similar characteristics, but the oocyte is without condensed chromatin within the nucleus and is often irregular in shape; the surrounding granulosa cells can be flat and pulled away from the edge of the follicle. A follicle of grade 3 contains a misshapen oocyte with or without nuclear vacuolation; theca and granulosa cells are separated from the edge of the follicle and the partly or fully disrupted granulosa have pyknotic nuclei [14]. Follicles of grades 1 and 2 were classified as normal and those of grade 3 as degenerated. Follicles were classified as degenerate if they had one or more of the following aspects: condensed oocyte nucleus; shrunken oocyte; nonhomogeneous ooplasm; pycnotic bodies in the granulosa cells; or low cellular density [15]. Examples of different follicular degenerations can be observed elsewhere [16, 17].

### Statistical analysis

Different rates of tissue after treatment was evaluated using ANOVA. Orthogonal contrasts were used to separate main effects. The level of statistical significance was set at a *P* <0.05.

## Results

After 45 days of development histological examination was conducted and only preantral (primordial, primary and secondary) follicles were viable. All the antral follicles in these two groups were degenerated and hence were not included in the counts.

**Fig. 2:**
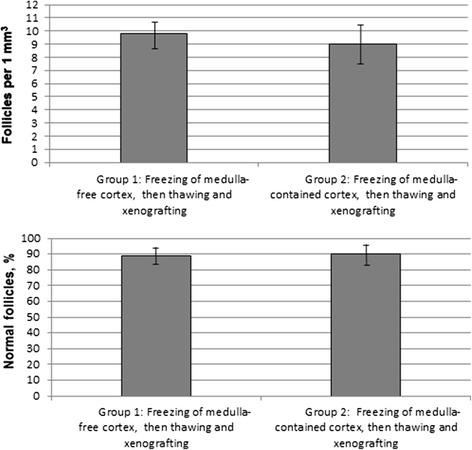
Effect of the containing of medula on the quality of follicles (expressed as quantity and normality of follicles). No statistical differences between respective rates (*P* > 0.1)

For Group 1 and Group 2, the mean densities of follicles per 1 mm^3^ were 9.8 ± 2.3, and 9.0 ± 3.1, respectively (*P* > 0.1). For these groups, 89.7 ± 5.2 and 90.1 ± 6.7 % preantral follicles were morphologically normal (*P* > 0.1) (Fig. 2).

**Fig. 3:**
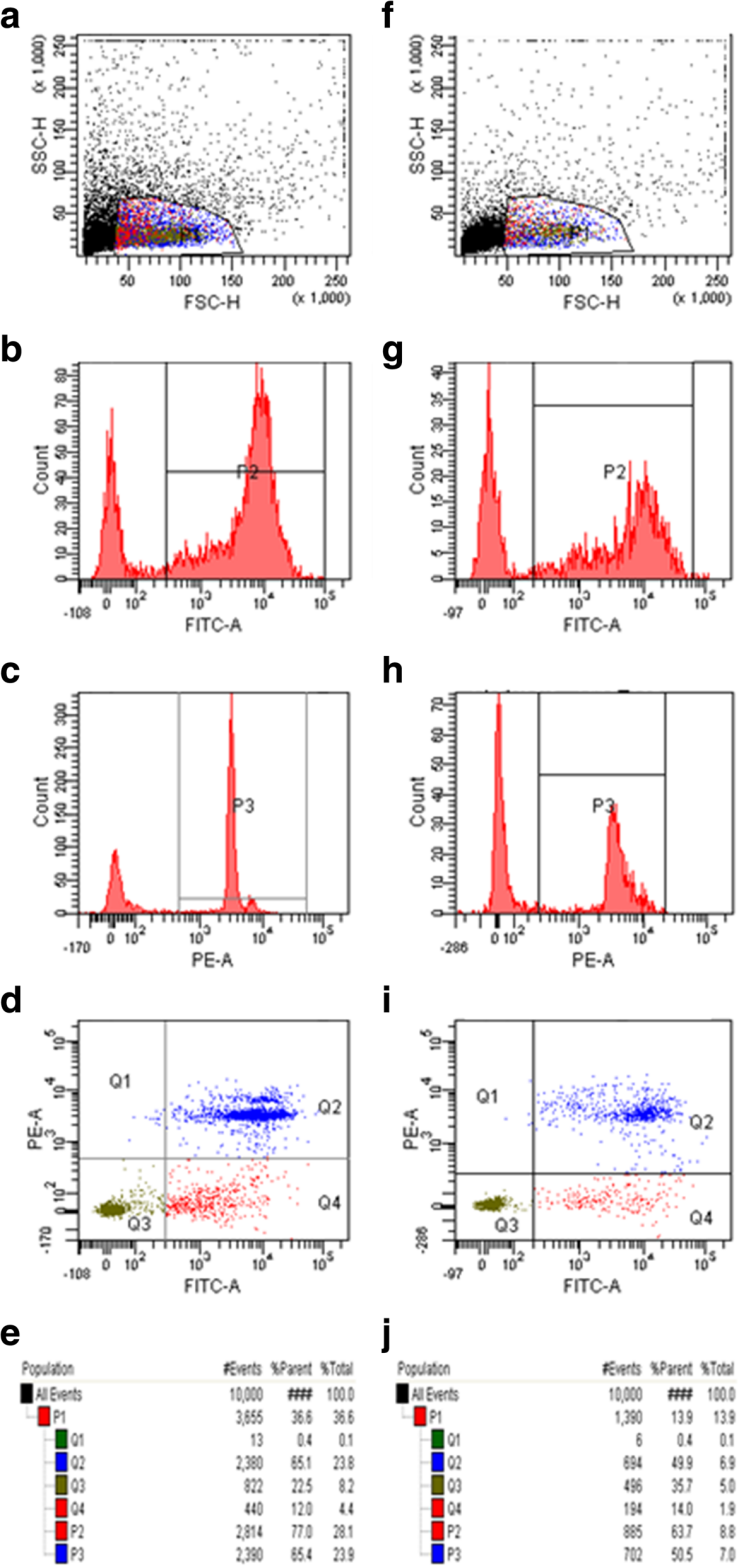
Translocation of phosphatidylserine in ovarian tissue frozen in form of medulla-free and medulla-contained strips and then xenografted in SCID mice: representative example of one experiment. **a**, **b**, **c**, **d**, **e** tissue frozen in form of medulla-free cortex, **f**, **g**, **h**, **i**, **j** tissue frozen in form of medulla-contained cortex, **a**, **f** forward and scatter dot plot used to select the interest population, **c**, **h** histograms displaying fluorescence of PE channel, used to measure fluorescence intensity of Propidium Iodide (PI), (D, I) dot plot analysis of FITC-Annexin V and PE channels, (Q1) cells negative to Annexin V (FITC A) and positive to PI (could indicate necrotic cells), (Q2) cells positive to both Annexin V and PI (could indicate late apoptotic stage), (Q3) cells negative for both Annexin V and PI (could indicate viable cells), (Q4) cells positive to FITC-Annexin V and negative to PI (could indicate early apoptotic state)

**Fig. 4:**
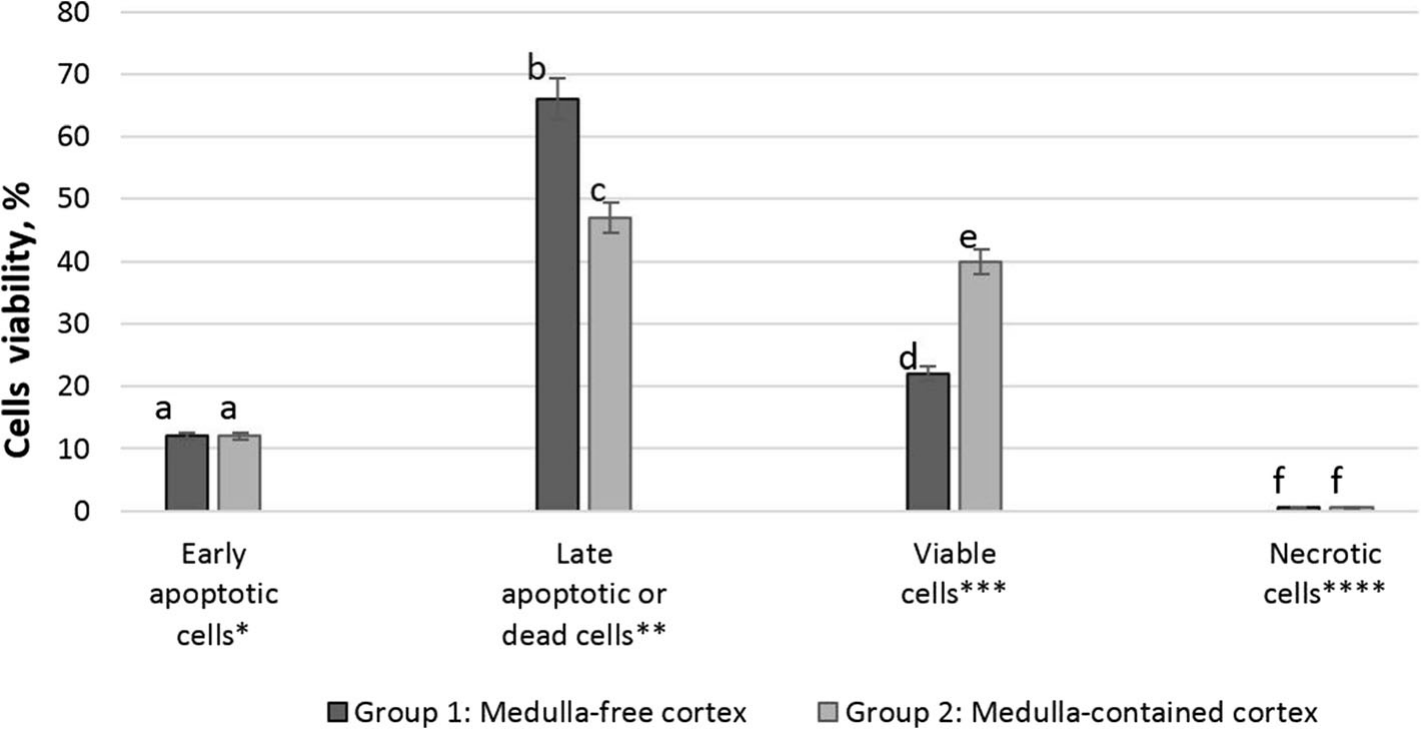
Translocation of phosphatidylserine in ovarian tissue frozen in form of medulla-free and medulla-contained strips and then xenografted in SCID mice. * Cells in early apoptotic state (FITC-Annexin V positive, PI negative). ** Cells in late apoptotic state or dead cells (FITC-Annexin V positive, PI positive). *** Viable cells (FITC-Annexin V negative, PI positive). **** Necrotic cells (FITC-Annexin V negative, PI positive). Different superscripts indicate statistical differences between respective rates (*P* < 0.05)

In Group 1 (ovarian tissue, which was frozen in form of medulla-free cortex) 12.4 ± 2.2 % of cells were in early apoptotic state (FITC-Annexin V positive, PI negative), while 65.6 ± 5.6 % of cells showed characteristics of late apoptotic state or dead cells (FITC-Annexin V positive, PI positive). In this group 21.6 ± 2.2 % were viable cells (FITC-Annexin V negative, PI positive) and 0.4 ± 0.05 % were necrotic cells (FITC-Annexin V negative, PI positive) (Figs. 3 and 4).

In Group 2 (ovarian tissue containing medulla) 12.5 ± 3.0 % of the cells were observed to be an early apoptotic state and 47.1 ± 3.1 % of the cells in a late apoptotic state. In this group 40.0 ± 4.2 % of the cells were viable and 0.4 ± 0.05 % of the cells were necrotic (Figs. 3 and 4).

## Discussion

In order to prevent crystallization of intracellular water at a cooling of mammalian ovarian tissues, permeable cryoprotectants are used to protect the cells. The main cryoprotectants are three glycols (high molecular alcohols: glycerol, propylene glycol and ethylene glycol) as well as dimethyl sulfoxide (DMSO) [18]. The ‘protective’ component is usually 10 to 12 % of the total salutation and is either made up from a single solution, often DMSO or a mixture of two cryoprotectants DMSO and one of the glycols [18]. In our protocol we used DMSO and ethylene glycol as we believe that ovarian tissue is multi-cellular organ and therefore requires for multi-cryprotectants in order to protect all types of cells. Furthermore, in a previous unpublished study we discovered that the effectiveness of 12 % DMSO alone was lower than a multi solution cryoprotectant in the same total concentration (12 %) (V. Isachenko, not published data).

The routine protocol of cryopreservation of ovarian tissue in our clinic presupposes the obvious long-time precooling of tissue before freezing. It is because we have previously established that the 24 h cooling to 5 °C before cryopreservation is beneficial for cryopreservation of human ovarian tissue [19].

During cryopreservation, at least 5 negative side effects have been observed: hypoxia, increasing of intracellular Ca^2+^, osmotic disruption of cellular membranes, generation of reactive oxygen species (ROS) and lipid peroxidation. Each can lead to translocation of phosphatidylserine.

Phosphatidylserine is the phospholipid component of membrane which plays a key role in cell cycle signaling, specifically in relationship to necrosis and apoptosis.

Phosphatidylserine is normally located on the inner side of the cell membrane. However, when a cell affected by some negative factors, phosphatidylserine is no longer restricted to the intracellular side of membrane and translocated to the extracellular surface of the cell, they act as a signal for macrophages to engulf the cells [8].

It has been noted that during cryopreservation, a cell initiates intracellular apoptotic or necrotic signaling in response to a stress. The binding of nuclear receptors by heat, hypoxia [20] and increased intracellular calcium concentration [21], can all trigger the release of intracellular apoptotic signals by a damaged cell. Apoptotic proteins that target mitochondria affect them in different ways. They can cause mitochondrial swelling through the formation of membrane pores, or they may increase the permeability of the mitochondrial membrane and cause apoptotic effectors to leak out [20].

Apoptosis is a major cause of sperm damage during cryopreservation [22]. It was established that in human ovarian tissue the percentage of apoptotic follicles was significantly higher in cryopreserved compared to fresh tissue at baseline (15 % vs. 4 %), indicating that cryopreservation process itself induces apoptosis in follicles [23].

In the early 1990s other research groups concluded that the translocation of phosphatidylserine played the role of detector of apoptosis [24]. However, it was later established that phosphatidylserine translocation may actually be reversible and also occurs in T-cell activation, without cell death [25].

Furthermore, it was found that phosphatidylserine translocation on the outer surface plasma membrane is less universal and more context dependent than previously thought [26]. Galluzzi et al. [26] published that using phosphatidylserine alone as a marker of early apoptosis, it should be noted that (1) if plasma membranes are permeabilized (as during late apoptosis or early necrosis) Annexin V can bind to intracellular phosphatidylserine; (2) phosphatidylserine translocation can prepare cells for phagocytic removal independently of apoptosis [27] and that (3) phosphatidylserine translocation can be compromised in cells in which autophagy is impaired [28].

As noted, a ‘specific’ cell death-related phenomenon may occur along with the execution of another cell death mode. For instance, excessive generation of reactive oxygen species (ROS) and reactive nitrogen species has been associated with several cases of apoptosis [29, 30]. Increased membrane permeability is a signal for early apoptosis in somatic cells [31].

When discussing our results we try to avoid the word ‘apoptosis’. As such, we evaluated the translocation of phosphatidylserine after respective cryo-treatment not as detector of apoptotic changes in cells but as a detector of viability of these cells after cryopreservation. By doing so we were able to determine the successes of various cryopreservation protocols. When the tissue was cryopreserved with the medulla there was a lower level of translocation compared to when the tissue was processed as cortex alone thereby allowing us to conclude that the medulla improves the freezing process.

In the number of publications the first stage in ovarian tissue cryopreservation has usually been the isolation of cortex from medulla [6]. This procedure allows the cryopreservation of primordial follicles, which are small and cryo-resistant structures [32, 33]. Usually slices of medulla-free cortex are prepared for cryopreservation. However, the ovarian medulla has two crucial characteristics: the presence of follicles and blood vessels. Follicle density in the medulla can reach 9824 follicles/g of medulla, and a considerable number of pre-antral follicles are lost when the medulla is discarded as required in current practice cortical tissue isolation prior to cryopreservation [9].

Therefore we attempted to answer if medulla-mediated promotion of neovascularization is advantageous for re-implanted ovarian cortex? And what advantage neo-vascularization may add to the preservation and/or development of follicles after transplantation?

The presence of blood vessels is essential for successful ovarian graft transplantation, required for the rapid establishment of the blood supply which is crucial for the survival of ovarian follicles [34]. It has been shown that transplanted immature rat ovaries become profusely re-vascularised within 48 h after autotransplantation [35]. In the cortex, development of primordial follicles is fully dependent on stromal vessels [36]. Prior to revascularisation, implants are vulnerable to ischemia, which is the main obstacle for the survival of tissue after transplantation. Such damage can lead to 30–70 % reduction in graft size accompanied with fibrotic changes [37]. The hypoxia observed during the first 5 days after grafting and ischemic damage occurring during this period could induce primordial follicle loss [38–40] and disorders of follicular activation [41, 42]. The presence of medullary material in ovarian cortex pieces could be expected to improve the chances of revascularization of transplanted ovarian tissue. It appears that the presence of medullar material in the ovarian pieces leads to better vascularization and is crucially needed for angiogenesis [43]. It can be inferred that inclusion of medulla would be of important advantage when such pieces are re-implanted into their female sources.

Comparative investigations of the role of medulla in human ovarian tissue are limited. Although, interesting comparative investigations in this field were performed by Revel et al., 2011 [44].

A 19-year-old woman had tissue from one of her ovaries cryopreserved prior to bone marrow transplantation, total body irradiation and sterilizing chemotherapy. As expected, premature ovarian failure resulted after treatment. Before cryopreservation of ovarian fragments, most of the ovarian medulla was removed and the resulting ovarian cortex tissue was manually cut into pieces varying in size from 0.8 to 1.4 cm in length, 2–4 mm in depth and 1.5–2.0 mm in width prior to cryopreservation [44].

Following thawing, eight pieces of ovarian cortex were transplanted. After ovarian transplantation, authors observed a decrease in FSH levels as well as an increase in estradiol. Authors noted that following three IVF cycles obtaining three poor quality embryos, the transplanted ovarian tissues stopped responding as ovulation induction failed to induce follicle development [44].

Later a second ovarian transplantation via laparoscopy revealed an atrophic left ovary with no follicles. The neo-ovarian tissue in the right broad ligament appeared to be vascularized yet no follicular structure was visible in the area where the tissue had been re-implanted. Biopsies of both sides were sent to histology and showed the presence of fibrotic tissue and no follicles. Authors created a peritoneal pocket, inserted thawed ovarian pieces into the right broad ligament and sutured the pocket. Following the second ovarian transplantation, authors again observed a decrease in FSH levels as well as an increase in estradiol [44]. However, it was noted that ovulation induction was not helpful. Four IVF cycles produced a total of six oocytes and two poor quality embryo transfers that failed to implant. Following repeat ovarian failure and no follicular response despite administration of maximal FSH doses, authors decided to modify the protocols before offering a third ovarian transplantation [44].

For the third ovarian transplantation, the 0.35 mm pieces were used. On the fourth IVF cycle, two mature oocytes were retrieved from the right transplanted tissue, yielding two good quality embryos, which were transferred and resulted in a pregnancy and live birth, which was achieved when thinner ovarian fragments without medulla were prepared prior to transplantation. As a result of the success, the authors concluded that making the fragments only a few hundred microns thick allows appropriate diffusion of gases and nutrients thereby preserving follicle viability.

Other research group describes separating the medulla from the cortex prior to tissue freezing [45]. However, they also recommend to do transplantation of up to 2 mm fragments which are comprised of both medulla and cortex and such the tissue is not in fact separated [45].

The following studies do indirectly support our conclusion that the presence of medulla in ovarian pieces is beneficial for post-thaw development of cryopreserved human ovarian tissue. The aim of this study was to determine the best source of isolated ovarian cells for the artificial ovary: cortex or medulla [46].

Ovarian tissue was retrieved from patients, and the ovarian cells from the cortex and medulla were isolated separately. Their viability was tested using a calcein AM/ethidium homodimer viability assay. Cells were then encapsulated in fibrin and grafted to peritoneal pockets in nude mice. Grafts recovered after 7 days were analyzed by immunohistochemistry for the presence of ovarian cells (Vimentin), proliferation (Ki67) and graft vascularization (double CD34). Cell apoptosis was analyzed by TUNEL assay [46].

Authors have concluded that ovarian cells from medullary tissue, which can be isolated in larger numbers, show higher viability and are able to improve graft vascularization [46].

## Conclusions

The presence of medulla in ovarian pieces is beneficial for post-thaw development of cryopreserved human ovarian tissue. For medical practice it is recommended to prepare ovarian medulla-contained strips.

## Abbreviations

FACS: Fluorescence-activated cell sorting
FITC: Fluorescein isothiocyanate (a chemical compound)
FSH: Follicle stimulating hormone
IU: International unites
PM: Revolutions per minute
ROS: Reactive oxygen species
SCID: Severe combined immunodeficiency
